# Effect Modification by Social Determinants of Pharmacogenetic Medication Interactions on 90-Day Hospital Readmissions within an Integrated U.S. Healthcare System

**DOI:** 10.3390/jpm12071145

**Published:** 2022-07-15

**Authors:** Loren Saulsberry, Lavisha Singh, Jaclyn Pruitt, Christopher Ward, Dyson T. Wake, Robert D. Gibbons, David O. Meltzer, Peter H. O’Donnell, Wanda Cruz-Knight, Peter J. Hulick, Henry M. Dunnenberger, Sean P. David

**Affiliations:** 1 Department of Public Health Sciences, The University of Chicago, Chicago, IL 60637, USA; rgibbons1@bsd.uchicago.edu; 2 Center for Personalized Therapeutics, The University of Chicago, Chicago, IL 60637, USA; podonnel@medicine.bsd.uchicago.edu; 3 Department of Biostatistics, NorthShore University HealthSystem, Evanston, IL 60201, USA; lsingh@northshore.org; 4 Outcomes Research Network, NorthShore University HealthSystem, Evanston, IL 60201, USA; jpruitt@northshore.org (J.P.); sdavid@northshore.org (S.P.D.); 5 Center for Personalized Medicine, NorthShore University HealthSystem, Evanston, IL 60201, USA; cward@northshore.org (C.W.); dwake@northshore.org (D.T.W.); phulick@northshore.org (P.J.H.); mdunnenberger@northshore.org (H.M.D.); 6 Section of Hospital Medicine, Department of Medicine, The University of Chicago, Chicago, IL 60637, USA; dmeltzer@medicine.bsd.uchicago.edu; 7 Committee on Clinical Pharmacology & Pharmacogenomics, The University of Chicago, Chicago, IL 60637, USA; 8 Department of Medicine, The University of Chicago, Chicago, IL 60637, USA; 9 Department of Family Medicine, NorthShore University HealthSystem, Evanston, IL 60201, USA; wcruz-knight@northshore.org; 10 Department of Medicine, NorthShore University HealthSystem, Evanston, IL 60201, USA

**Keywords:** pharmacogenetics, personalized medicine, primary care, social determinants of health (SDoH)

## Abstract

The present study builds on our prior work that demonstrated an association between pharmacogenetic interactions and 90-day readmission. In a substantially larger, more diverse study population of 19,999 adults tracked from 2010 through 2020 who underwent testing with a 13-gene pharmacogenetic panel, we included additional covariates to evaluate aggregate contribution of social determinants and medical comorbidity with the presence of identified gene-x-drug interactions to moderate 90-day hospital readmission (primary outcome). Univariate logistic regression analyses demonstrated that strongest associations with 90 day hospital readmissions were the number of medications prescribed within 30 days of a first hospital admission that had Clinical Pharmacogenomics Implementation Consortium (CPIC) guidance (CPIC medications) (5+ CPIC medications, odds ratio (OR) = 7.66, 95% confidence interval 5.45–10.77) (*p* < 0.0001), major comorbidities (5+ comorbidities, OR 3.36, 2.61–4.32) (*p* < 0.0001), age (65 + years, OR = 2.35, 1.77–3.12) (*p* < 0.0001), unemployment (OR = 2.19, 1.88–2.64) (*p* < 0.0001), Black/African-American race (OR 2.12, 1.47–3.07) (*p* < 0.0001), median household income (OR = 1.63, 1.03–2.58) (*p* = 0.035), male gender (OR = 1.47, 1.21–1.80) (*p* = 0.0001), and one or more gene-x-drug interaction (defined as a prescribed CPIC medication for a patient with a corresponding actionable pharmacogenetic variant) (OR = 1.41, 1.18–1.70). Health insurance was not associated with risk of 90-day readmission. Race, income, employment status, and gene-x-drug interactions were robust in a multivariable logistic regression model. The odds of 90-day readmission for patients with one or more identified gene-x-drug interactions after adjustment for these covariates was attenuated by 10% (OR = 1.31, 1.08–1.59) (*p* = 0.006). Although the interaction between race and gene-x-drug interactions was not statistically significant, White patients were more likely to have a gene-x-drug interaction (35.2%) than Black/African-American patients (25.9%) who were not readmitted (*p* < 0.0001). These results highlight the major contribution of social determinants and medical complexity to risk for hospital readmission, and that these determinants may modify the effect of gene-x-drug interactions on rehospitalization risk.

## 1. Introduction

Hospital readmissions is an important indicator of quality within a health care system, which is monitored nationally [1,2,3]. In the U.S., one study found more than one in five hospital readmissions were due to adverse drug reactions (ADRs) [4]. Patients with multiple comorbidities (i.e., health conditions) and frequent hospitalizations are at increased cumulative risk for potential adverse drug responses (ADRs) compared to other groups [5,6]. Furthermore, underrepresented and underserved patient populations who are more likely to experience chronic health conditions and poorer health outcomes, such as African Americans, have been shown to be at greater risk for mortality due to ADRs [5,7]. At present, how medical and social determinants of health (SDoH) collectively impact risk for ADRs across different healthcare settings is unclear.

Pharmacogenomics is a type of personalized and precision medicine that can avoid ADRs and more efficiently optimize drug treatment by incorporating patient genetic information associated with individual variation in drug response into prescribing decisions. A gene-x-drug interaction exists when there is at least one genetic association that could guide prescribing. A systematic review of preemptive pharmacogenetic genotyping evaluated its impact on hospital admissions and whether it prompts medication changes [8]. This review found that patients receiving pharmacogenomic testing compared to those without testing experienced significantly fewer all-cause hospitalizations and that medication changes producing patient benefit were more frequent. However, concerns exist that implementing pharmacogenomics into clinical settings might introduce/exacerbate health disparities that have been well-documented in the U.S. health system [5]. Prescription medications are administered frequently during hospitalizations presenting an opportunity to modify/optimize drug treatment for patients, especially those that may have less access to the health care system and fewer health encounters with prescribing providers. In particular, one study within an African-American patient cohort showed that the time of hospital discharge may present a window of opportunity to evaluate prescribed pharmacogenomic medications and intervene to prevent adverse drug events amongst patient populations more likely to experience health disparities [9]. Related to pharmacogenomics implementation into clinical practice, it remains heavily debated whether race/ethnicity, which is frequently recorded in EHR’s, can appropriately proxy the range of experiences within and beyond the health system that might inform medical care and improve minority health [10,11,12,13].

Previous evaluations of the associations between pharmacogenetic phenotypes (i.e., drug response) and hospital admissions reported mixed results [14,15], yet these studies did not evaluate gene-x-drug interactions identified through genetic testing. In a recent analysis, the presence of one or more gene-x-drug interactions with medications associated with guidance from the Clinical Pharmacogenomics Implementation Consortium (CPIC) increased the risk of hospital readmissions [16]. That same study also demonstrated that compared to patients with no chronic conditions, patients with multiple comorbidities who were prescribed a medication with CPIC guidance within 30 days of an initial inpatient hospital stay were more than two times more likely to experience a hospital readmission within 90 days. To date, research has focused less on comprehensively evaluating the roles of health-related sociodemographic risk factors and complex medical needs (e.g., multiple comorbidities) in modifying the effects of gene-x-drug interactions and hospital readmissions.

The current study had several aims. The first was to assess the relationship between gene-x-drug interactions and risk of 90-day hospital readmission in a greatly enlarged patient study population that was double the size of prior preliminary analyses. The second was to more comprehensively account for a range of social determinants that may impact this relationship with additional consideration of the influence of factors such as insurance status, income, and multiple comorbidities. Finally, we aimed to evaluate with more granularity the impact of race/ethnicity, socioeconomics, and comorbidity on the modification of the relationship between gene-x-drug interactions and risk of 90-day hospital readmission. We hypothesized that social determinants were important contributors to readmission and that there would be effect modification of the gene-x-drug interactions on risk of 90-day readmission, particularly by medical complexity.

## 2. Materials and Methods

### 2.1. Study Setting and Population

**Figure 1:**
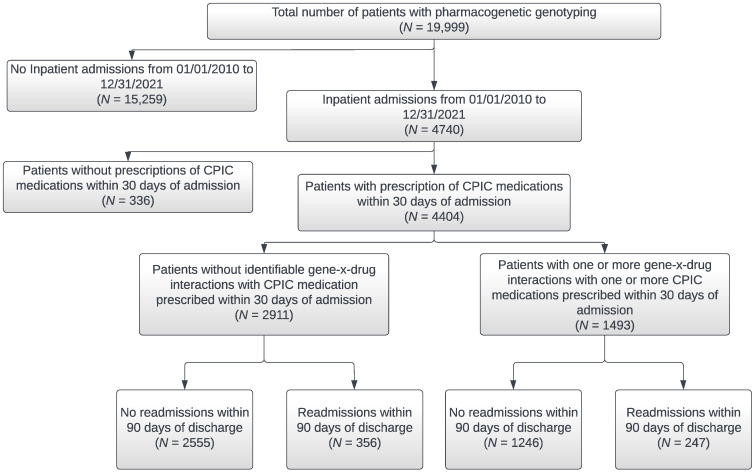
Flow Diagram of Patient Selection for Analyses.

The study setting was the NorthShore University HealthSystem, which is an integrated healthcare delivery system including six hospitals and a multispecialty group practice (NorthShore Medical Group) with more than 140 locations in the Chicagoland area. NorthShore has approximately 60,000 annual inpatient admissions, 100,000 ER visits, and three million outpatient encounters annually. The study population included patients ages 18 or older who were clinically tested through December 2021 for 13 genes with associated Clinical Pharmacogenetics Implementation Consortium (CPIC) guidelines with actionable clinical recommendations. The genotyping method varied based on the clinical offering at the time of testing and the majority of patients were tested as part of a large population screening program using a next generation sequencing (NGS) Color ™ (Burlingame, CA, USA) panel that also included cardiology and hereditary cancer related genes [16,17]. The remaining patients were genotyped with a NGS panel by Sema4 ™ (Stamford, CT, USA) (*n* = 3607), by the Center for Personalized Medicine using laboratory developed assays (*n* = 2191), or by a third-party vendor (*n* = 6). In this study, a total of 20,090 patients were genotyped, but our inclusion criteria included adults ages 18 or older, leaving a final study population of 19,999 patients. All patients included in the study provided consent to care along with written informed consent to have their clinical data utilized for operational purposes. Test results were reported in the electronic health record (EHRs) and accessible to patients’ primary care physicians and clinicians across the health system. All hospital admissions from January 2010 through December 2020 were collected from electronic health records. However, in order to capture 90-day readmissions data collection regarding these identified hospital admissions extended through March 2021. Other information gathered from EHR data included medications prescribed within 30 days of hospital admission that had associated CPIC guidelines with evidence levels A-B for the pharmacogenes that were genotyped (i.e., CPIC medications), patient demographics, major chronic disease conditions, smoking status, and COVID-19 status. Age, sociodemographic variables, insurance status, and number of CPIC prescription medications represent the most recent available data current as of March 2022. The specific types of insurance comprising the insurance status categories including “Commercial,” “Government,” and “Out-of-pocket (self-pay)” are defined in the Supplementary Materials, Table S1. Figure 1 is a flow diagram of patients included in the analyses based on hospital admission and gene-x-drug interactions.

### 2.2. Data Collection

Data was extracted from EHRs (EPIC, EpiCare software, February 2022 update, Verona, WI, USA) for the study period from 1 January 2010 to 31 December 2020 inclusive of all admissions, with 90-day readmissions captured through 31 March 2021. CPIC medications prescribed to patients within 30 days prior to their first inpatient hospital stay during the study period and through the date of that initial hospitalization discharge were recorded. A total of 44 CPIC medications with evidence levels A-B for the pharmacogenes were recorded in this study. These CPIC medications included amitriptyline, atazanavir, atomoxetine, azathioprine, capecitabine, celecoxib, citalopram, clomipramine, clopidogrel, codeine, desipramine, doxepin, efavirenz, escitalopram, fluorouracil, flurbiprofen, fluvoxamine, fosphenytoin, ibuprofen, imipramine, lansoprazole, lornoxicam, meloxicam, mercaptopurine, nortriptyline, omeprazole, ondansetron, pantoprazole, paroxetine, peginterferon alfa-2a/2b, phenytoin, piroxicam, sertraline, simvastatin, tacrolimus, tamoxifen, tenoxicam, thioguanine, tramadol, trimipramine, tropisetron, voriconazole, and warfarin. Additional information on these CPIC medications, their associated genes, and their clinical relevance can be found in the Supplementary Materials, Table S2. For genes with evidence of variation in drug response, genetic indicators (genotype-based phenotypes) were collected and annotated, for *CYP2B6*, *CYP2C19*, *CYP2C9*, *CYP2D6*, *CYP3A5*, *CYP4F2*, *DPYD*, *IFNL3*, *NUDT15*, *SLCO1B1, TPMT, UGT1A1, VKORC1*. The frequency with which these medications were prescribed to patients within 30 days of an initial hospital admission within our study population can also be found in the Supplementary Materials, Table S2 for the overall study population as well as stratified by race, ethnicity, and rehospitalization history.

Patients (*n* = 4404) with at least one inpatient hospital admission from 2010 to 2020 were included in regression analyses to evaluate the primary outcome of 90-day hospital readmissions.

Patient sociodemographic variables including age, gender, race, ethnicity, marital status, employment status, insurance status, and median household income were also collected. Median household income from 2016 to 2020 was downloaded from data source American Community Survey (Table B19013) using Metopio [18]. The income information was matched to the patients using zip codes. Clinical patient characteristics extracted from EHRs included body mass index (BMI), smoking status, COVID-19 test results, chronic health conditions, and number of comorbidities. The chronic health conditions documented included history of cancer, chronic obstructive pulmonary disease (COPD), type 1 or 2 diabetes, myocardial infarction (MI), heart failure (HF), peripheral vascular disease (PVD), asthma, or stroke/cerebrovascular accident (CVA). Patients were defined as having a gene-x-drug interaction if they were prescribed at least one CPIC medication within 30 days of their first inpatient hospital admission and had a corresponding actionable pharmacogenetic variant with prescribing guidance for that variant. The gene-x-drug interaction designation does not necessarily indicate incongruence between the CPIC recommended dose or drug selection recommendation and the prescribed dose or drug.

### 2.3. Descriptive Analyses

Sociodemographic and clinical variables were compared between patients with 90-day readmissions and no hospital readmissions using a Wilcoxon rank sum test for continuous variables and a Chi-square or Fisher’s exact test for categorical variables (Table 1). Patient descriptive statistics for continuous variables were reported as median, the overall range, and the interquartile range (IQR). For categorical variables, frequency and percentage were presented. Bivariate relationships between sociodemographic variables were further explored for comparison of patients with 90-day readmissions and no hospital readmissions (Table 2). Finally, the presence/absence of gene-x-drug interactions by race/ethnicity were compared between patients with 90-day readmissions and no hospital readmissions (Table 3). Patient descriptive statistics for continuous variables were reported as median, the overall range, and the interquartile range (IQR). For categorical variables, frequency and percentage were presented.

### 2.4. Statistical Analyses

Univariate and multivariable logistic regression analyses were performed to determine the association between the main independent variable of pharmacogenomic medication interactions and the primary outcome of 90-day hospital readmission. Multivariable logistic regression models were adjusted for variables with a *p*-value less than 0.1 in univariate analysis and/or variables theorized to be important sociodemographic and clinical predictors of 90-day hospital readmission. The primary outcome of interest was 90-day hospital readmissions, and the main independent variable was one or more identified gene-x-drug interactions. The covariates included for adjustment included age, sex, race, employment status, insurance status, income, body mass index (BMI), smoking status, and the number of comorbidities. Although the number of CPIC medications was included in univariate analyses, this variable was not included in the multivariable logistic regression model because it is highly correlated with both the likelihood by chance of a gene-x-drug interaction and the outcome. Odds ratios (ORs) and corresponding 95% confidence intervals (CIs) are reported. *p*-values less than 0.05 were considered significant. All statistical analyses were performed using SAS version 9.4 (SAS Institute Inc., Cary, NC, USA).

## 3. Results

### 3.1. Overall Study Population Sociodemographic and Clinical Characteristics

The study population included 19,999 patients for the analyses in total. Table 1 includes the sociodemographic and clinical characteristics of the study population. Almost one in four patients experienced at least one hospital inpatient admission from 1 January 2010 to 31 December 2020. Older adults (65 + years, 32.1%), females (27.5%), Black/African American patients (28.6%), and unemployed individuals (31.4%) were more likely to experience a hospitalization at least once over this 11-year study period. With each additional comorbidity, patients had an increased likelihood of experiencing an inpatient hospital admission. Of the 4740 patients that had at least one inpatient hospital admissions, 13% were readmitted to the hospital within 90 days. Increased age was also associated with a higher likelihood of 90-day hospital readmission (*p* < 0.0001). While female patients were more likely to experience an earlier inpatient hospital admission, males had a higher likelihood of having a hospital readmission within 90 days (*p* < 0.0001). A higher proportion of Black/African American (25.1%) patients were readmitted to the hospital within 90 days compared to any other racial group of patients, and they were almost two times more likely to be readmitted to the hospital than White patients (13.5%). Just as unemployment and multiple comorbidities were associated with increased likelihood of at least one inpatient hospital admission, these patient factors also were related to a higher chance of 90-day hospital readmission.

Of the 19,999 patients, 15,213 (76%) were prescribed at least one medication with corresponding CPIC prescribing guidance (“CPIC medications”) at least once between 2010 and 2020. Among the 4740 patients admitted to the hospital, 4404 (93%) were prescribed at least one medication with CPIC pharmacogenetic guidance. There was a direct relationship between the number of CPIC medications prescribed within 30 days of an initial hospital admission and likelihood of 90-day hospital readmissions such that each additional CPIC medication prescribed increased patient likelihood for rehospitalization (*p* < 0.0001). Amongst patients prescribed at least one of these medications within 30 days of an initial hospital admission, patients with at least one gene-x-drug interaction compared with patients with no identified gene-x-drug interaction were more likely to experience a hospital readmission (16.5% vs. 11.5%, *p* < 0.0001).

### 3.2. Characteristics of Patient Subpopulations with Inpatient Admissions and 90-Day Hospital Readmissions

Table 2 presents the rate of inpatient admissions and 90-day hospital readmissions for patient subpopulations. While the majority of the study population had median household incomes above the national median ($64,994), Black patients with incomes below (25.5%) and above (29.8%) the national median were significantly more likely (*p* < 0.0001) than White patients at similar income levels (9.8% and 13.7%, respectively), to be readmitted to the hospital. Whether prescribed only one or more than five CPIC medications within 30 days of hospitalization, White patients were more likely than Black patients to experience an initial inpatient hospital admission. However, among patients prescribed at least two CPIC medications, Black patients were about twice as likely than White patients with similar exposure to CPIC medications to experience hospital readmission within 90 days.

### 3.3. Presence of at Least One Gene-x-Drug Interaction by Race/Ethnicity and 90-Day Hospital Readmissions

Table 3 presents the presence/absence of gene-x-drug interactions by race/ethnicity for patients with 90-day readmissions and no hospital readmissions. Among group with no readmissions, more than one in three White patients (35.2%) had an identified gene-x-drug interaction compared to 25% of Black/African American patients who had an identified gene-x-drug interaction. Similar proportions of within each patient race group experienced 90-day readmissions (White 41.8%; Black/African American 41.5%; Asian 41.2%).

### 3.4. Effect Modification of Patient Sociodemographic and Clinical Characteristics on 90 Day Hospital Readmissions

Table 4 presents results of univariate and multivariable logistic regression of risk of 90-day hospital readmission (primary outcome) for patients prescribed at least one CPIC medication within 30 days of an initial hospital admission. Older patients (65 + years) (OR = 2.35, 95% CI 1.77–3.12) (*p* < 0.0001), males (OR = 1.47, 95% CI 1.21–1.80) (*p* = 0.0001), Black/African American patients (OR = 2.12, 95% CI 1.47–3.07) (*p* < 0.0001), employment status (OR = 2.19, 95% CI 1.82–2.64) (*p* < 0.0001), the presence of three or more comorbidities (OR = 3.36, 95% CI 2.61–4.32) (*p* < 0.0001), or number of CPIC medication prescriptions (five vs. one) (OR = 7.66, 95% CI 5.45–10.77) (*p* < 0.0001) were also associated with higher rates of 90 day hospital readmission. A dose-response effect was observed with number of patient comorbidities as a larger number of comorbid conditions was associated with higher odds of 90-day readmissions; this effect observed in univariate analyses was attenuated following adjustment. Alternatively, Black/African American patients compared to White patients remained at higher odds for 90 day hospital readmissions following adjustment for sociodemographic and comorbid clinical conditions (OR = 2.12, 95% CI 1.42–3.17) (*p* < 0.0001). Though patients with health insurance provided by a government entity (see Supplementary Material, Table S1 for further details) were more likely to report initial inpatient hospital admissions (34.5%, *p* < 0.0001) and 90-day hospital readmissions (19%, *p* < 0.0001) compared to patients with commercial insurance or who paid health expenses out-of-pocket, insurance status was not a significant predictor of 90-day hospital readmissions. In this study’s substantially larger study population, the unadjusted odds of 90-day readmission were more than 40% higher (OR = 1.41, 95% CI 1.18–1.70) (*p* = 0.0002) for patients with a gene-x-drug interaction than patients without an identified gene-x-drug interaction. After adjustment for this study’s expanded set of sociodemographic and clinical factors, the odds of hospital readmission for patients with at least one gene-x-drug interaction compared to no gene-x-drug interaction were slightly attenuated but remained statistically significant (OR = 1.31, 95% CI 1.08–1.59) (*p* = 0.006).

Sensitivity analyses (not shown) were performed using multiple logistic regression to further investigate whether an interactive effect between race and having an identified gene-x-drug interaction could impact the outcome of 90-day hospital readmissions. The interaction term was not found to be statistically significant (*p* = 0.205). After adjusting for interaction effects and other covariates from the full model, the odds of hospital readmission for patients with at least one gene-x-drug interaction compared to no gene-x-drug interaction remained statistically significant (OR = 1.24, 95% CI 1.02–1.52) (*p* = 0.035).

## 4. Conclusions

### 4.1. Study Conclusions

In this study, we evaluated the influence of patient sociodemographic and clinical characteristics on the relationship between identified gene-x-drug interactions and risk of 90-day hospital readmission. Our results indicate that patient-level factors including being of an older age (65 + years), male, Black/African American, unemployed, having multiple comorbidities, and the presence of an identified gene-x-drug interaction were independent drivers of 90-day hospital readmissions. Insurance status and income were not significant predictors of 90-day hospital readmissions. In this study population, which was doubled in size compared to prior analyses, the presence of gene-x-drug interactions was a significant predictor of 90-day hospital readmission, increasing patient risk of rehospitalization by 40%. Collectively accounting for patient sociodemographic and clinical traits attenuated this effect by 10%. Black/African American patients were consistently about two times more likely than White patients to experience hospital readmissions within 90 days of discharge from an initial inpatient hospital stay even after adjusting for sociodemographic and clinical factors. For patients that experienced readmissions within 90 days, about four out of every ten patients regardless of race had an identified gene-x-drug interaction. However, the frequency of having an identified gene-x-drug interaction varied among patients of different racial backgrounds for those with no rehospitalizations during the study period with the highest proportion of White patients (35.2%) found to have gene-x-drug interactions.

Notably, the effect sizes for the relationship between the presence of gene-x-drug interactions and 90-day hospital readmissions in this study with a larger sample size evaluating a more comprehensive set of social determinants, replicated earlier findings. The current study provides additional evidence that having a gene-x-drug interaction increases risk for deleterious health outcomes, such as hospital readmissions, but patient pharmacogenomic associations represent only one piece of the picture in risk assessment. Patient social determinants of health also offer insights into assessing risk for rehospitalization. Furthermore, our results align with earlier findings that Black Medicare patients have higher hospital readmissions rates even after controlling for multiple patient-level factors [19,20,21]. Previous indicators of healthcare access in these studies, such as insurance status and income, did not play a significant role in predicting readmissions in the current study. The full underlying drivers of these disparities in readmissions are not yet understood, and additional research is needed to comprehend the contribution of specific medical and social determinants of health on hospital readmissions, especially for different racial/ethnic populations. This study provides some evidence that the prevalence of multiple comorbidities and a greater number of prescribed medications, which are both indicators of medical complexity for patients with significant health needs, may explain some of the variation in readmissions. Indeed, we observed a decrease in the odds of 90-day readmissions once our model adjusted for the number of comorbidities. In addition, descriptive analyses of the number of comorbidities by race illustrate higher proportions of Black/African American patients compared to White patients reported having one or more comorbidities. This indicates that a higher prevalence of comorbid conditions amongst Black/African American patients may be one piece of the puzzle in completing the full picture of what factors are driving their higher rates of readmissions.

Interestingly, in our study, patients experiencing readmissions within 90 days had similar frequencies of gene-x-drug interactions regardless of their race. It is noteworthy though that in our study the proportion of White patients who were not readmitted and who had at least one identified gene-x-drug interaction was higher than for Black/African-American patients. This observation could, speculatively, be explained by more than one factor. The sensitivity of standard pharmacogenetic panels to variants expressed more prevalently in non-European ancestry patients may be lower than for European-ancestry patients, reflecting the fact that most of the genetic markers “defined” as actionable by CPIC were derived in European descent populations [22,23]. It is also possible that some patients who experienced side effects related to gene-x-drug interactions may have had different levels of medication adjustments depending on disparities in access or other structural barriers to care. We cannot say, definitively, that there were systematic or structural inequities in pharmacogenomic health services or pharmacovigilance, because in sensitivity analyses, the race by gene-x-drug interaction was not a statistically significant predictor of 90-day hospital readmissions. Nonetheless, hospitalization may be a particularly vulnerable time for the most economically and socially vulnerable patients. Hospital discharge could therefore be a high-value clinical setting for pharmacogenomics implementation, as it may provide intervention opportunities for vulnerable patients with health complexities or challenges [24], especially those at greater risk to experience health disparities. While the potential impact of pharmacogenomics could be significant to avoiding ADRs and improving drug treatment for underrepresented, underserved, and/or marginalized populations that experience health-related social vulnerability and have fewer encounters with the healthcare system, pharmacogenomic studies related to scientific discovery and implementation persistently underrepresent populations of diverse genetic ancestry [22,23]. Further research is needed to better understand how pharmacogenomics can be tailored and equitably implemented in minority patient populations across healthcare settings to reduce health disparities.

### 4.2. Study Limitations

This study has several limitations. Since the data were not curated for medication dose and its relationship to evidence-based dosing recommendations for a given genotype, we were unable to evaluate clinical adherence to CPIC prescribing guidelines amongst prescribing healthcare providers. The specific adverse events that could trigger hospital readmission and outcomes were not possible to examine with the available data. Another limitation is that we were unable to untangle the degree to which the observed associations between gene-x-drug interactions was driven by the number of CPIC medications prescribed regardless of the presence of one or more pharmacogenetic interactions, or if pharmacogenetic interactions conferred additional, incremental risk for 90-day hospital readmission. Many other factors that affect drug response and ADRs were not captured in the available EHR data and could not be accounted for in multivariable modeling, such as pharmacogenetic phenotypes and other health-related social factors that could affect management of health conditions. Finally, due to the way the data are collected in the EHR, we could not fully assess the degree of social vulnerability patients of different backgrounds were experiencing within our study population. The magnitude of these challenges to health maintenance and avoiding hospitalization may be vital to risk assessment.

### 4.3. Study Implications

Despite these limitations, our study contributes to filling a gap in the literature by lending insights into the significant influence of these socioeconomic factors on the relationship between the presence of gene-x-drug interactions and 90-day hospital readmissions for a study population prescribed one or more medications associated with evidence for genetic variability in drug response. In particular, these results strongly suggest that social determinants are important contributors to readmission and that there is effect modification of the gene-x-drug interactions on risk of 90-day readmission, particularly by medical complexity. The subsequent reduction in risk following adjustment for social determinants indicate that a more holistic approach to the implementation of pharmacogenomics within a hospital setting accounting for both the medical and social determinants of health may reduce risk of adverse patient health outcomes.

## Figures and Tables

**Table 1:** Overall Study Population Sociodemographic and Clinical Characteristics (*n* = 19,999).

*n* (%) √	Inpatient Admissions 2010–2020 ^a^						
	Overall 19,999 (100)	No 15,259 (76)	Yes 4740 (24)		90-Day Hospital Readmission ^b^		
					*n* = 4740		
					No 4120 (87)	Yes 620 (13)	
**Age, median (IQR)**	53 (42–64)	53 (42–63)	54 (41–68)	*	52 (41–67)	62 (46–72)	*
**Age group (years)**							
18–39	4097 (20.5)	3128 (76.3)	969 (23.7)		877 (90.5)	92 (9.5)	
40–49	4275 (21.4)	3208 (75)	1067 (25)		976 (91.5)	91 (8.5)	
50–64	6849 (34.2)	5679 (82.9)	1170 (17.1)		1011 (86.4)	159 (13.6)	
65 or above	4778 (23.9)	3244 (67.9)	1534 (32.1)		1256 (81.9)	278 (18.1)	
**Gender**				*			*
Female	12,852 (64.3)	9324 (72.5)	3528 (27.5)		3106 (88)	422 (12)	
Male	7147 (35.7)	5935 (83)	1212 (17)		1014 (83.7)	198 (16.3)	
**Race**				*			*
White	13,961 (69.9)	10,292 (73.7)	3669 (26.3)		3172 (86.5)	497 (13.5)	
Black/African American	612 (3.1)	437 (71.4)	175 (28.6)		131 (74.9)	44 (25.1)	
Asian	1293 (6.5)	1020 (78.9)	273 (21.1)		254 (93)	19 (7)	
American Indian/Alaska Native	37 (0.2)	28 (75.7)	9 (24.3)		8 (88.9)	1 (11.1)	
Pacific Islander/Hawaiian Native	18 (0.1)	16 (88.9)	2 (11.1)		2 (100)	0 (0)	
Other	3751 (18.8)	3157 (84.2)	594 (15.8)		537 (90.4)	57 (9.6)	
**Ethnicity**				*			
Hispanic/Latino	981 (4.9)	758 (77.3)	223 (22.7)		196 (87.9)	27 (12.1)	
Non-Hispanic	18,653 (93.4)	14,159 (75.9)	4494 (24.1)		3902 (86.8)	592 (13.2)	
**Marital status**				*			*
Unmarried	5700 (28.5)	4472 (78.5)	1228 (21.5)		1010 (82.2)	218 (17.8)	
Married	14,123 (70.6)	10,620 (75.2)	3503 (24.8)		3102 (88.6)	401 (11.4)	
**Employment status**				*			*
Employed	12,961 (64.8)	10,309 (79.5)	2652 (20.5)		2405 (90.7)	247 (9.3)	
Unemployed	6328 (31.6)	4338 (68.6)	1990 (31.4)		1624 (81.6)	366 (18.4)	
**Insurance status**				*			*
Commercial	16,387 (81.9)	12,851 (78.4)	3536 (21.6)		3144 (88.9)	392 (11.1)	
Government	3425 (17.1)	2244 (65.5)	1181 (34.5)		957 (81)	224 (19)	
Out-of-pocket (self-pay)	187 (0.9)	164 (87.7)	23 (12.3)		19 (82.6)	4 (17.4)	
Median Household income in relation to US median household income ***							
Above 64,994	18,634 (93.3)	14,198 (76.2)	4436 (23.8)		3849 (86.8)	587 (13.2)	
64,994 or less	1330 (6.7)	1030 (77.4)	300 (22.6)		268 (89.3)	32 (10.7)	
**BMI, median (range, 13.3–79.2)**	27.0 (23.7–31.2)	26.8 (23.7–30.8)	27.6 (23.9–32.3)	*	27.5 (23.8–32.1)	28.6 (24.3–33.5)	*
**Smoking status**							
No	17,854 (96.6)	13,349 (74.8)	4505 (25.2)		3918 (87)	587 (13)	
Yes	632 (3.4)	478 (75.6)	154 (24.4)		127 (82.5)	27 (17.5)	
**COVID Status**							
Yes	176 (0.9)	130 (73.9)	46 (26.1)		41 (89.1)	5 (10.9)	
Number of comorbidities ****				*			*
0	11,972 (64.8)	9398 (78.5)	2574 (21.5)		2334 (90.7)	240 (9.3)	
1	4170 (22.6)	3074 (73.7)	1096 (26.3)		946 (86.3)	150 (13.7)	
2	1197 (6.5)	749 (62.6)	448 (37.4)		360 (80.4)	88 (19.6)	
3 or more	1147 (6.2)	606 (52.8)	541 (47.2)		405 (74.9)	136 (25.1)	
**Comorbidities**							
Cancer	225 (1.2)	112 (49.8)	113 (50.2)	*	82 (72.6)	31 (27.4)	*
COPD	226 (1.2)	99 (43.8)	127 (56.2)	*	92 (72.4)	35 (27.6)	*
Diabetes	1378 (7.5)	851 (61.8)	527 (38.2)	*	412 (78.2)	115 (21.8)	*
History of Diabetes	1518 (8.2)	930 (61.3)	588 (38.7)	*	461 (78.4)	127 (21.6)	*
Myocardial Infarction	129 (0.7)	41 (31.8)	88 (68.2)	*	72 (81.8)	16 (18.2)	
Heart Failure	247 (1.3)	91 (36.8)	156 (63.2)	*	101 (64.7)	55 (35.3)	*
Hypertension	4211 (22.7)	2861 (67.9)	1350 (32.1)	*	1093 (81)	257 (19)	*
PVD	219 (1.2)	97 (44.3)	122 (55.7)	*	96 (78.7)	26 (21.3)	*
Asthma	1745 (9.4)	1195 (68.5)	550 (31.5)	*	456 (82.9)	94 (17.1)	*
CVA	547 (3)	257 (47)	290 (53)	*	212 (73.1)	78 (26.9)	*
**CPIC medications prescribed within 30 days prior to inpatient admission (*n* = 4404)**					**3801/4404 (86.3)**	**603/4404 (13.7)**	
**1 or more gene-x-drug interactions with 1 or more CPIC medications prescribed within 30 days prior to inpatient admission**							*
Absent					2874 (88.5)	373 (11.5)	
Present					1246 (83.5)	247 (16.5)	
**Number of CPIC medications prescribed within 30 days prior to inpatient admission**							*
1					777 (92.4)	64 (7.6)	
2					1749 (92)	152 (8)	
3					620 (83.6)	122 (16.4)	
4					390 (80.7)	93 (19.3)	
5 or more					265 (60.6)	172 (39.4)	

^a^ Inpatient hospital admissions between 1 January 2010 to 31 December 2020 inclusive. ^b^ Readmissions within 90 days of inpatient admission discharge date between 1 January 2010 and inclusive of 90-day readmissions through 31 March 2021. * *p*-values less than 0.05. *** The newly released 2016–2020 ACS 5-year data shows that U.S. median household income increased to $64,994 when compared to the 2011–2015 ACS 5-year data adjusted for inflation. **** Comorbidities include Cancer, COPD, Diabetes, Diabetes PL/HX, Myocardial Infarction, Heart failure, Hypertension, PVD, Asthma, and CVA. Chronic obstructive pulmonary disease (COPD). Peripheral vascular disease (PVD). Cerebrovascular Accident (CVA). CPIC: Clinical Pharmacogenetics Implementation Consortium (https://cpicpgx.org/, accessed on 18 June 2022). CPIC medications prescribed up to 30 days prior to inpatient admission date through date of hospital discharge in electronic health record between 1 December 2009 and 31 December 2020. Unknown/missing values not shown. **√** Proportions presented in the table do not include missing values for the variable.

**Table 2:** Characteristics of Patient Subpopulations with Inpatient Admissions and 90-day Hospital Readmissions.

	Inpatient Admissions from 1 January 2010 to 31 December 2020						
	Overall	No	Yes		Yes		
					4740		
					90-Day Hospital Readmissions		
					No	Yes	
*n* (%)	19,999	15,259 (76)	4740 (24)	*p*-Value	4120 (87)	620 (13)	*p*-Value
**Race by US median household income**				0.32			**<0.0001**
White and below US median income	706 (4.9)	512 (72.5)	194 (27.5)		175 (90.2)	19 (9.8)	
White and above US median income	13,241 (91)	9769 (73.8)	3472 (26.2)		2995 (86.3)	477 (13.7)	
Black and below US median income	137 (0.9)	102 (74.5)	35 (25.5)		28 (80)	7 (20)	
Black and above US median income	466 (3.2)	327 (70.2)	139 (29.8)		102 (73.4)	37 (26.6)	
**Number of comorbidities by race**				**<0.0001**			**<0.0001**
0 and White	8316 (60.3)	6391 (76.9)	1925 (23.1)		1737 (90.2)	188 (9.8)	
0 and Black	268 (1.9)	199 (74.3)	69 (25.7)		56 (81.2)	13 (18.8)	
1 and White	3164 (23)	2254 (71.2)	910 (28.8)		786 (86.4)	124 (13.6)	
1 and Black	150 (1.1)	112 (74.7)	38 (25.3)		29 (76.3)	9 (23.7)	
2 and White	891 (6.5)	538 (60.4)	353 (39.6)		283 (80.2)	70 (19.8)	
2 and Black	60 (0.4)	30 (50)	30 (50)		20 (66.7)	10 (33.3)	
3+ and White	850 (6.2)	414 (48.7)	436 (51.3)		325 (74.5)	111 (25.5)	
3+ and Black	81 (0.6)	47 (58)	34 (42)		22 (64.7)	12 (35.3)	
**Number of comorbidities by US median household income**				**<0.0001**			**<0.0001**
0 and ≤ $64,994 (below median)	700 (3.8)	563 (80.4)	137 (19.6)		126 (92)	11 (8)	
0 and > $64,994 (above median)	11,248 (61)	8813 (78.4)	2435 (21.6)		2207 (90.6)	228 (9.4)	
1 and ≤ $64,994 (below median)	279 (1.5)	203 (72.8)	76 (27.2)		65 (85.5)	11 (14.5)	
1 and > $64,994 (above median)	3884 (21)	2865 (73.8)	1019 (26.2)		880 (86.4)	139 (13.6)	
2 and ≤ $64,994 (below median)	79 (0.4)	47 (59.5)	32 (40.5)		29 (90.6)	3 (9.4)	
2 and > $64,994 (above median)	1117 (6.1)	701 (62.8)	416 (37.2)		331 (79.6)	85 (20.4)	
3+ and ≤ $64,994 (below median)	100 (0.5)	54 (54)	46 (46)		40 (87)	6 (13)	
3+ and > $64,994 (above median)	1046 (5.7)	552 (52.8)	494 (47.2)		364 (73.7)	130 (26.3)	
**Number of CPIC medications prescribed within 30 days before admission date by race**							**<0.0001**
1 and White			639 (17.9)		589 (92.2)	50 (7.8)	
1 and Black			30 (0.8)		29 (96.7)	1 (3.3)	
2 and White			1375 (38.5)		1258 (91.5)	117 (8.5)	
2 and Black			62 (1.7)		49 (79)	13 (21)	
3 and White			606 (17)		507 (83.7)	99 (16.3)	
3 and Black			28 (0.8)		20 (71.4)	8 (28.6)	
4 and White			407 (11.4)		328 (80.6)	79 (19.4)	
4 and Black			18 (0.5)		11 (61.1)	7 (38.9)	
5+ and White			390 (10.9)		247 (63.3)	143 (36.7)	
5+ and Black			19 (0.5)		7 (36.8)	12 (63.2)	
**Number of CPIC medications prescribed within 30 days before admission date by US median household income**							**<0.0001**
1 and ≤ $64,994 (below median)			57 (1.3)		53 (93)	4 (7)	
1 and > $64,994 (above median)			782 (17.8)		723 (92.5)	59 (7.5)	
2 and ≤ $64,994 (below median)			111 (2.5)		105 (94.6)	6 (5.4)	
2 and > $64,994 (above median)			1789 (40.7)		1643 (91.8)	146 (8.2)	
3 and ≤ $64,994 (below median)			40 (0.9)		37 (92.5)	3 (7.5)	
3 and > $64,994 (above median)			702 (16)		583 (83)	119 (17)	
4 and ≤ $64,994 (below median)			34 (0.8)		27 (79.4)	7 (20.6)	
4 and > $64,994 (above median)			448 (10.2)		362 (80.8)	86 (19.2)	
5+ and ≤ $64,994 (below median)			31 (0.7)		23 (74.2)	8 (25.8)	
5+ and > $64,994 (above median)			406 (9.2)		242 (59.6)	164 (40.4)	

Unknown/missing values not shown.

**Table 3:** Identified Gene-x-Drug Interactions by Race/Ethnicity and 90-day Hospital Readmissions.

	No 90-Day Readmission			90-Day Readmission		
CPIC Medications Ordered within 30 Days of Inpatient Admission (*n* = 4404)	*n* = 3801			*n* = 603		
1 or more Gene-x-Drug Interactions	Absent	Present		Absent	Present	
*n* (%)	2555 (67)	1246 (33)	*p*-Value	356 (59)	247 (41)	*p*-Value
**Race**			**<0.0001**			0.70
White	1898 (64.8)	1031 (35.2)		284 (58.2)	204 (41.8)	
Black or African American	86 (74.1)	30 (25.9)		24 (58.5)	17 (41.5)	
Asian	196 (81.7)	44 (18.3)		10 (58.8)	7 (41.2)	
American Indian or Alaska Native	6 (75)	2 (25)		1 (100)	0 (0)	
Pacific Islander/Hawaiian Native	1 (50)	1 (50)		0 (0)	0 (0)	
Other	355 (72.3)	136 (27.7)		35 (64.8)	19 (35.2)	
**Ethnicity**			0.12			0.12
Hispanic/Latino	133 (72.7)	50 (27.3)		20 (76.9)	6 (23.1)	
Non-Hispanic	2405 (66.9)	1192 (33.1)		335 (58.2)	241 (41.8)	

Unknown/missing values not shown.

**Table 4:** Unadjusted and Adjusted Logistic Regression Analysis of 90-day Hospital Readmissions Among Patients Prescribed a CPIC Medication Within 30 days of Inpatient Admission (*n* = 4404).

uOR (95% CI)		*p*-Value	aOR (95% CI)	*p*-Value
**Age Group**				
18–39	*Reference*		*Reference*	
40–49	1.05 (0.75–1.47)	0.001	1.04 (0.74–1.47)	0.529
50–64	1.73 (1.27–2.35)	0.026	1.28 (0.93–1.78)	0.104
65 or above	2.35 (1.77–3.12)	<0.0001	1.14 (0.80–1.65)	0.783
**Gender**				
Female	*Reference*		*Reference*	
Male	1.47 (1.21–1.80)	0.0001	1.11 (0.89–1.38)	0.371
Race *				
White	*Reference*		*Reference*	
Black/African American	2.12 (1.47–3.07)	<0.0001	2.12 (1.42–3.17)	<0.0001
Asian	0.43 (0.26–0.70)	<0.0001	0.62 (0.37–1.03)	0.002
**Ethnicity**				
Non-Hispanic	*Reference*			
Hispanic/Latino	1.10 (0.54–2.26)	0.975		
**Marital status**				
Unmarried	*Reference*		-	-
Married	0.60 (0.49–0.72)	0.441		
Employment status **				
Employed	*Reference*		*Reference*	
Unemployed	2.19 (1.82–2.64)	<0.0001	1.74 (1.39–2.18)	0.007
**Insurance status**				
Commercial	*Reference*		*Reference*	
Government	1.81 (1.50–2.20)	0.307	1.04 (0.81–1.34)	0.6605
Out-of-pocket (self-pay)	1.67 (0.47–5.90)	0.736	1.48 (0.41–5.32)	0.5742
Median household income in relation to US median household income ***				
Less than $64,994	*Reference*		*Reference*	
$64,994 or more	1.33 (0.86–2.05)	0.203	1.63 (1.03–2.58)	0.035
**BMI, range 13.3–79.2**	1.03 (1.02–1.04)	<0.0001	1.01 (1.00–1.03)	0.050
**Smoking status**				
No	*Reference*		*Reference*	
Yes	1.38 (0.87–2.18)	0.176	1.26 (0.78–2.04)	0.347
**COVID status**				
Yes vs. No	0.56 (0.17–1.84)	0.341		
Number of comorbidities ****				
0	*Reference*		*Reference*	
1	1.57 (1.24–1.98)	0.018	1.28 (0.99–1.64)	0.044
2	2.54 (1.91–3.39)	0.006	1.83 (1.33–2.52)	0.070
3 or more	3.36 (2.61–4.32)	<0.0001	2.23 (1.66–3.02)	<0.0001
**Number of CPIC medications prescribed within 30 days prior to inpatient admission**				
1	*Reference*			
2	1.14 (0.82–1.58)	<0.0001	-	-
3	2.50 (1.77–3.53)	0.417	-	-
4	3.07 (2.13–4.41)	0.008	-	-
5 or more	7.66 (5.45–10.77)	<0.0001	-	-
**Gene-x-drug interactions within 30 days prior to inpatient admission**				
Absent	*Reference*		*Reference*	
Present	1.41 (1.18–1.70)	0.0002	1.31 (1.08–1.59)	0.006

uOR: Unadjusted Odds Ratio, aOR: Adjusted Odds Ratio, CI: Confidence Interval. Reference: rows including “*Reference*” refer to the main category of the variables that the others are compared to (e.g., male (comparator) vs. female (reference)). Admission dates from electronic health record 1 December 2009 and 31 December 2020 inclusive. Results reflect unadjusted and adjusted logistic regression models. Logistic regression model adjusted for age, sex, race, employment status, insurance status, income, body mass index (BMI), smoking status, number of comorbidities, and gene-x-drug interactions. * Excluding race “other” and “unknown.” ** Employed includes full time, part time, and self-employed; Unemployed includes not employed, retired, and students. *** The newly released 2016–2020 ACS 5-year data shows that U.S. median household income increased to $64,994 when compared to the 2011–2015 ACS 5-year data adjusted for inflation. **** Comorbidities include Cancer, Chronic Obstructive Pulmonary Disorder (COPD), Diabetes, Diabetes PL/HX, Myocardial Infarction, Heart failure, Hypertension, Peripheral Vascular Disease (PVD), Asthma, and Cerebrovascular Accident (CVA). *p*-values less than 0.02 were considered statistically significant.

## Data Availability

Data relevant to the study have been included in the article. Further data are available from the authors upon request.

## Supplementary Materials

The following supporting information can be downloaded at: https://www.mdpi.com/article/10.3390/jpm12071145/s1, Table S1: Patient insurance status by type; Table S2: CPIC medications prescribed by race/ethnicity for patients with and without 90-day readmissions.
